# A review on architecture with fungal biomaterials: the desired and the feasible

**DOI:** 10.1186/s40694-021-00124-5

**Published:** 2021-11-19

**Authors:** Dimitra Almpani-Lekka, Sven Pfeiffer, Christian Schmidts, Seung-il Seo

**Affiliations:** 1 Wühlischstr. 26, 10245 Berlin, Germany; 2 Chair of Digital Design, Planning and Building, Department of Architecture, Bochum University of Applied Sciences, Am Hochschulcampus 1, 44801 Bochum, Germany; 3 Chair of Digital and Experimental Design, Department of Architecture and Urban Planing, University of the Arts, Hardenbergstr. 33, 10623 Berlin, Germany; 4 TU Berlin, Fraunhoferstraße 25, 10587 Berlin, Germany

**Keywords:** Architecture, Bio materials, Digital planning processes, Fungal architecture

## Abstract

Fungal biomaterials are becoming increasingly popular in the fields of architecture and design, with a significant bloom of projects having taken place during the last 10 years. Using mycelium as a stabilizing compound for fibers from agricultural waste, new building elements can be manufactured according to the circular economy model and be used for architectural construction to transform the building industry towards an increased environmental and economic sustainability. Simultaneously, research on those materials and especially fungal biocomposites is producing knowledge that allows for the materials themselves to inspire and transform the architectural design. Novel research on those materials is not only allowing for their use as construction materials, but it inspires and affects the architectural design process through the discovery and variation of the materials’ properties. Today, many interdisciplinary teams are working on this emerging field to integrate fungal biocomposites in the construction industry and to merge science, art, and architecture responsibly.

This study provides an overview of the progress that has been made in this field during the last 10 years, focusing on six works that are presented in more detail. Those six works are spaces at an architectural scale which showcase unique elements and innovative aspects for the use of fungal biomaterials in architecture. Each work has followed different design strategies, different fabrication methods, or different post-processing methods. All of them together have produced significant technical knowledge as well as a cultural impact for the field of architecture but also for the field of fungal biotechnology.

## Introduction

**Fig. 1:**
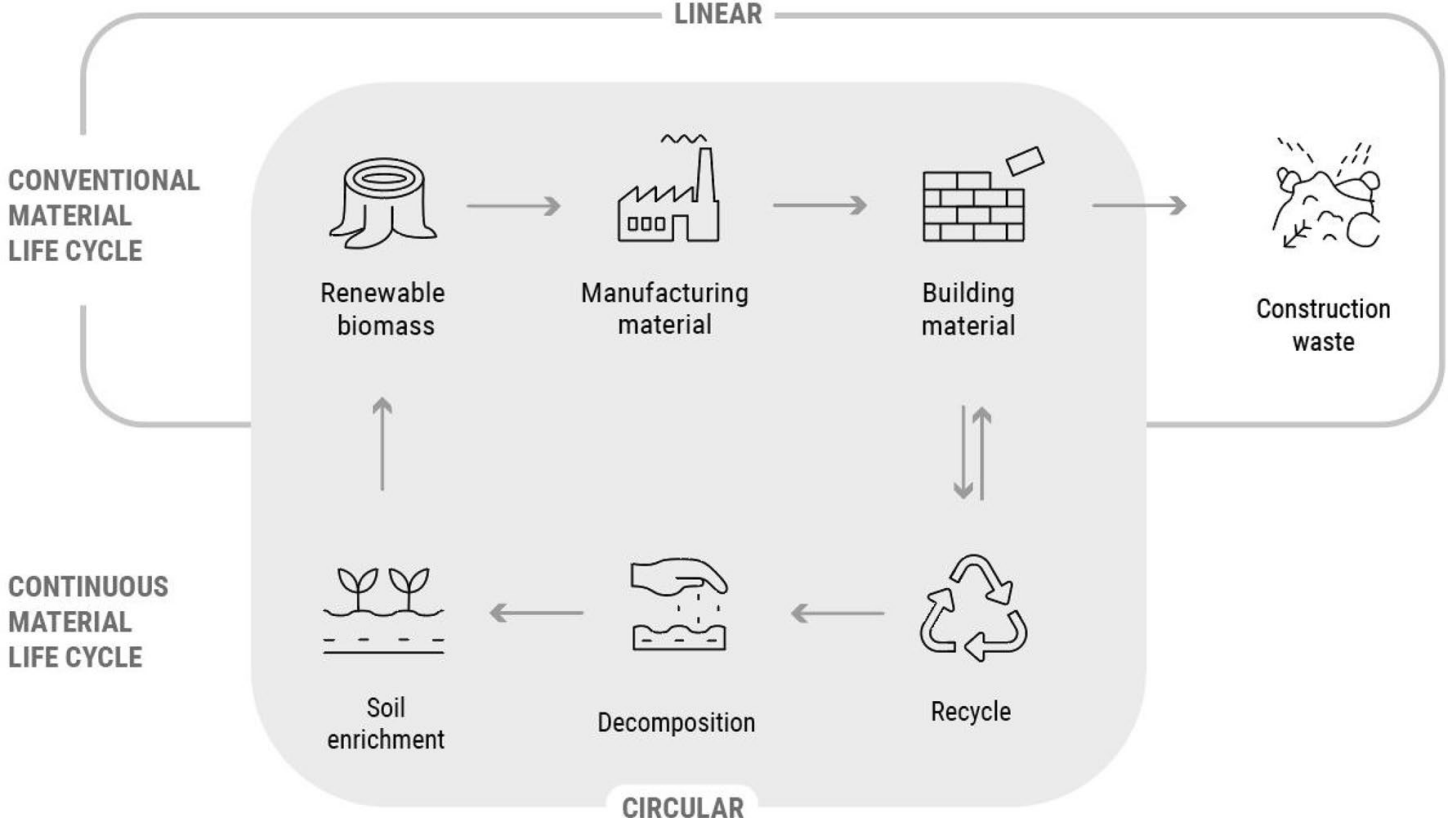
Diagram comparing linear/circular building processes

During the last 10 years the fields of architecture and design have shown a considerate interest in transforming their practices towards a more environmentally and socially sustainable model. According to the United Nation’s ‘Global Status Report for Buildings and Construction’ of 2020, the building sector is responsible for 38% of all energy-related CO_2_ emissions, including building construction industry emissions [1]. To reduce this number, the architecture and the construction industry is currently searching for holistic solutions that provide increased energy efficiency of the construction process, and buildings that are integrated and adaptive elements of the environment. To that end, the focus is shifting towards the research, design and application of innovative ecological construction materials that follow the principles of circular economic and energy flows throughout their life cycle. Whereas conventional building processes start with obtaining raw materials from nature and ending with their disposal, continuous building life cycles use low-impact materials which can be re-introduced as source for new building activities (Fig. 1).

In this endeavor, architecture over the last years is showing a considerable turn towards material technology research on efficient biomaterials that are produced with minimal carbon footprints and are biodegradable or compostable. The design and production of such materials for architectural purposes are the result of efforts from interdisciplinary teams that include material scientists, chemists, architects, and biotechnologists among others. With the use of biotechnological methods of production, these materials span from bioplastics to flexible membranes and fungal bio-composites, and are used for furniture, as soundproofing and thermal insulation panel systems, stacking modular elements (bricks), flooring tiles and many more [2]. Particularly, during the last 10 years a great interest has been shown towards fungal architecture to harness the properties of mycelium as a base material, to explore its different characteristics and develop original applications for it. The members of the interdisciplinary teams that work on this emerging field inform, enhance, and learn from each other’s practices through collaboration. Specifically, their work entails the extraction of information about the mechanical behavior of fungal materials, the analysis and understanding of their aesthetic qualities and the creation of concepts about new ways in which they can be used. The morphological and philosophical investigations of fungi and their mycelium, combined with a thorough understanding of their physical properties pave the ground for architects to visualize an alternative future for the built environment. This extends beyond the materials and construction methods that we follow, to new ways we can experience our buildings and new morphologies and typologies that can emerge from this fascinating culmination of fields. In this paper, we will overview some important works of fungal biotechnological applications in architecture, focusing on the ones that use mycelium as a construction material. We will briefly analyze and categorize those works by specific criteria and will proceed to talk about significant breakthroughs and difficulties that emerged during their realization. Finally, we will close with the importance these works have within the architectural context and what impact they have on the possibilities and challenges of the future of fungal architecture and the transformation of architecture into an environmentally conscious practice.

## Characteristic works of fungal architecture and construction—a summary of the current state of the field

**Fig. 2:**
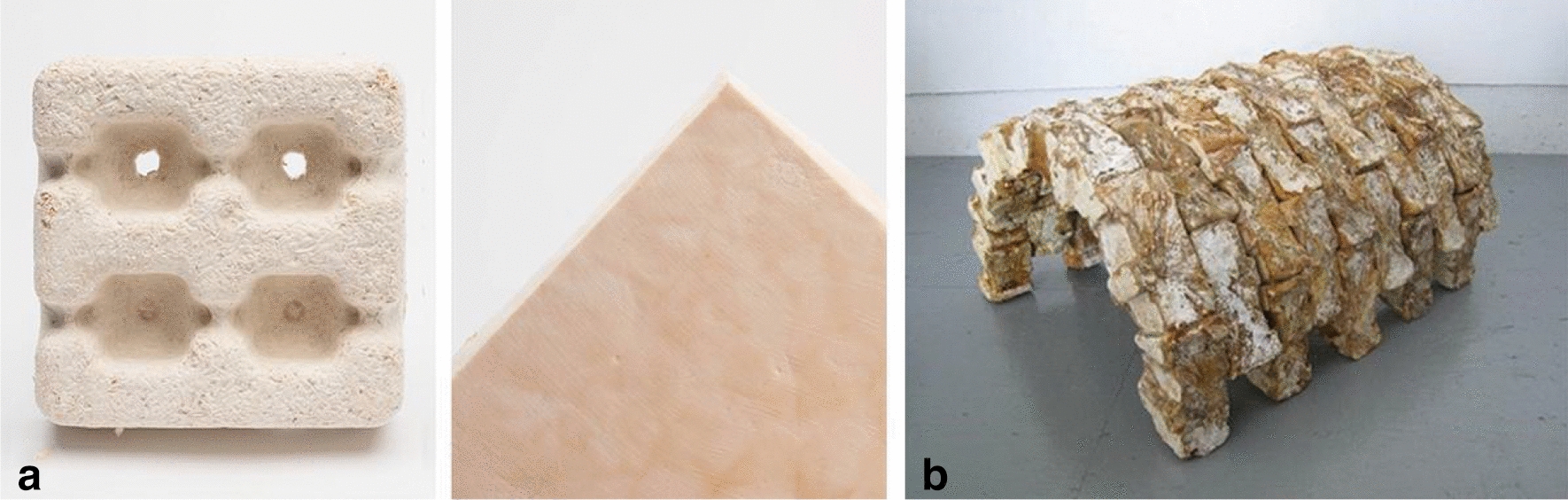
**a** The products from Ecovative Design. [5] Credit: © Ecovative Design. **b** ‘Mycotecture Vault’ (2011). [6] Credit: © Phil Ross

The field of fungal architecture and design is currently under rapid development. According to AECOM’s building sustainability expert David Cheshire, mycelium materials can be ‘part of the solution’ to carbon-negative buildings [3]. The current state of fungal architecture has been shaped largely by the work of companies such as ‘Ecovative’ whose co-founders developed and patented several methods of fabricating mycelium products as substitutes for conventional packaging materials, insulation boards, food products, etc. [4] (Fig. 2a). However, artists Phil Ross and Sophia Wang who co-founded the company Mycoworks, brought the use of fungi in buildings at the forefront of architecture. Their 2011 exhibition series ‘Mycotecture’ included vaults, wall segments and different structural installations made from interlocking bricks of mycelium composites (Fig. 2b), showcasing the wide potential this material has for construction [7]. Over the last 10 years, new projects using this material are appearing ever more frequently.

Although this paper is focusing on the use of mycelium as a construction material, it is important to mention that fungi and microbes in general are influencing architecture and design in many more aspects. Architects and urbanists working on digital and computational design are analyzing and ‘decoding’ microscopic structures like mycelium’s hyphae network to learn from the geometric, morphological, and interactive gestures of microorganisms. This creates a field where architects can explore the ‘growth algorithms’ of such structures and translate or simulate them with the use of digital tools into intelligent designs and resilient urban infrastructures [8].

## Classification of presented works

As eligibility criteria for the selected case studies we firstly considered the use of mycelium based biocomposites as primary construction materials for the realization of architectural projects. Excluded are object design and art projects, furniture, as well as commercially available paneling and insulation items that are supplemental. The selected projects contributed at the time of their completion new design approaches, uses of the material, manufacturing processes, fabrication methods and production scales to the field of fungal architecture. We followed a chronological order of presenting the works and excluded later examples that covered the same characteristics mentioned above. For the composition of this paper we acquired information from peer reviewed articles published by the creators of the projects, as well as articles and interviews published by prestigious architecture and engineering journals. Our critical review on the works also relies on our own experience and involvement with this topic. The reviewed examples are classified in Table 1. The classification system specifies the environment of the constructions, the type of mycelium component used, the fungal strain used, the substrate mixture, the supporting structure, and the post-treatment. All the projects stand as complete architectural proposals that have been created with digital design tools, although using different approaches and design strategies.

**Table 1:**
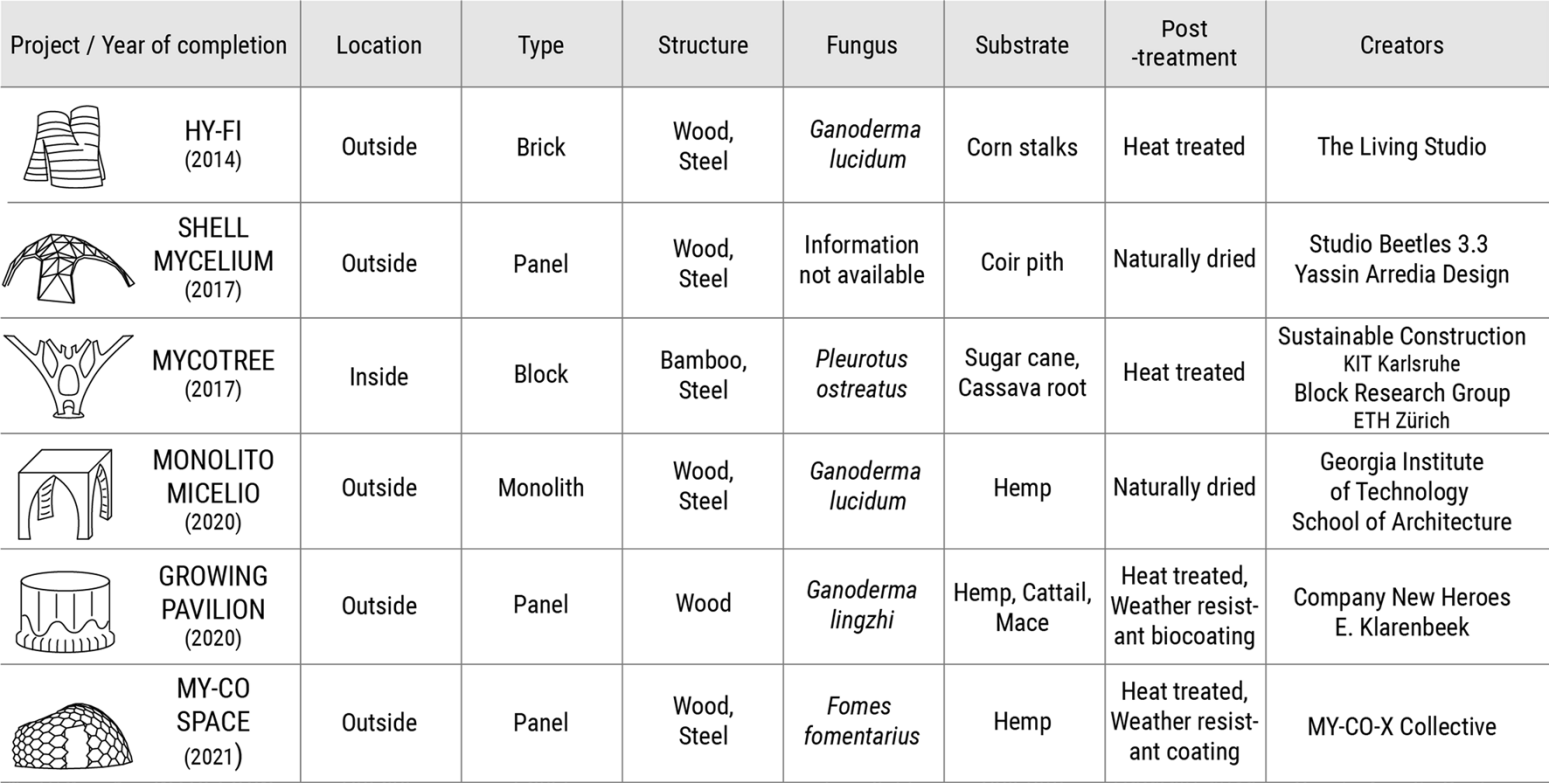
Comparison of reviewed projects

**Hy-Fi (2014)**, The Living/D. Benjamin and Arup.

The Hy-Fi pavilion was built in the MoMa PS1 art museum in New York. Ecovative created mycelium-composite blocks that could be used in a similar way to bricks of a masonry wall. Around 10,000 of these blocks were used in this pavilion, making Hy-Fi the largest construction project with mycelium composite materials to date. This project expressed the possibility of using the material in a modular manner and already existing methods of construction. The geometry of this pavilion is designed as a cluster of merging cylinders that provides shade and ensures cooling through interior updrafts of air. Gaps have been left between the bricks for controlled ventilation. The top of the pavilion was coated with a special light refraction film developed by 3 M. The structure of the pavilion was anchored with reusable ground screws on hempcrete bricks that were used for the foundation. The company ‘Arup’ who did the structural analysis for the pavilion found that the bricks could carry their weight in this height (13 m) and withstand over 65mph of wind gusts. However, in order to minimize wind caused movement, they decided to maintain the supporting scaffolding planks of the construction forms [9]. After the exhibition, the bricks were shredded and dispersed on soil. 60 days later they were degraded [10].

**Shell Mycelium (2016)**, Studio Beetles 3.3/A. Rahman and Yassin Arredia Design/G. Arredia, M. Yassin.

**Fig. 3:**
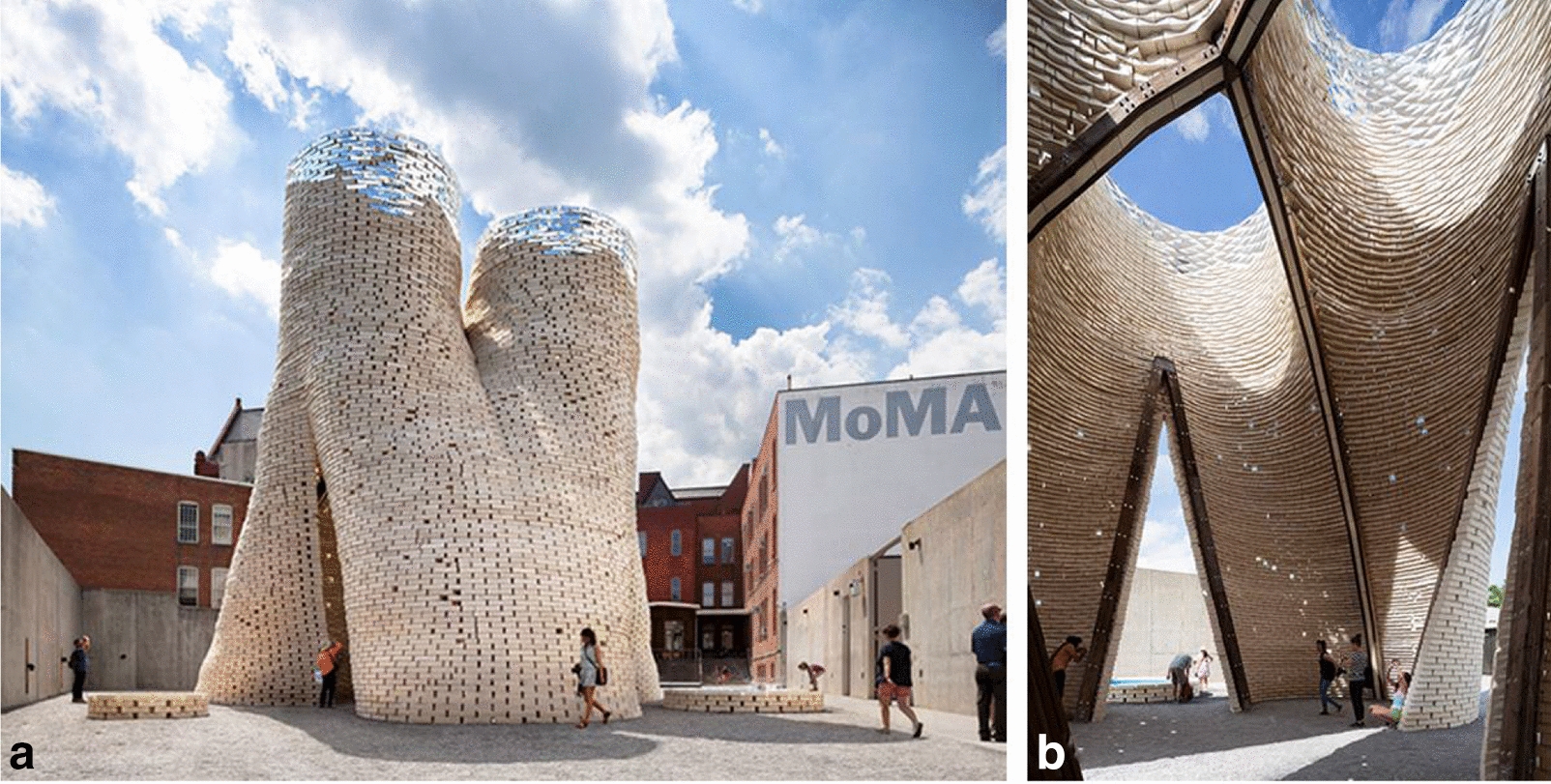
**a** Exterior Perspective of the pavilion, **b** Exploded diagram of the different construction layers. Credit: © BEETLES 3.3 and Yassin Areddia Designs / Photographs by Krishna & Govind Raja [11]

This pavilion (Fig. 3a) was designed by Studio Beetles 3.3 and Yassin Arredia Design for the 2016 Kochi-Muziris Biennale in Southwest India. The goal was to produce a dismantlable and lightweight structure that could be used for temporary events. A wooden grid shell was used as the load-bearing structure. The substrates were placed in the cavities of each plywood frame on top of the pavilion and were inoculated with mycelium (Fig. 3b). Steel connectors were used between the truss elements. One of the main features of this pavilion is that the mycelium-substrate mixture was not pre-grown in sterile conditions, but rather allowed to grow on-site in open air. The designers intended to also let the mycelial components dry naturally by sunlight exposure. During the Biennale, a thin layer of mycelium started covering the composite, but the composite dried out naturally before binding fully [11]. This project was an informative attempt at a non-discreet use of the mycelium composite and showcased what the challenges to such an approach are.

**MycoTree (2017)**, Sustainable Construction KIT Karlsruhe/K. Schlesier, F. Heisel, and D. Hebel; Block Research Group ETH Zürich/J. Lee et al.; Alternative Construction Materials, Future Cities Laboratory, Singapore -ETH Centre: N. Saeidi, et al.

**Fig. 4:**
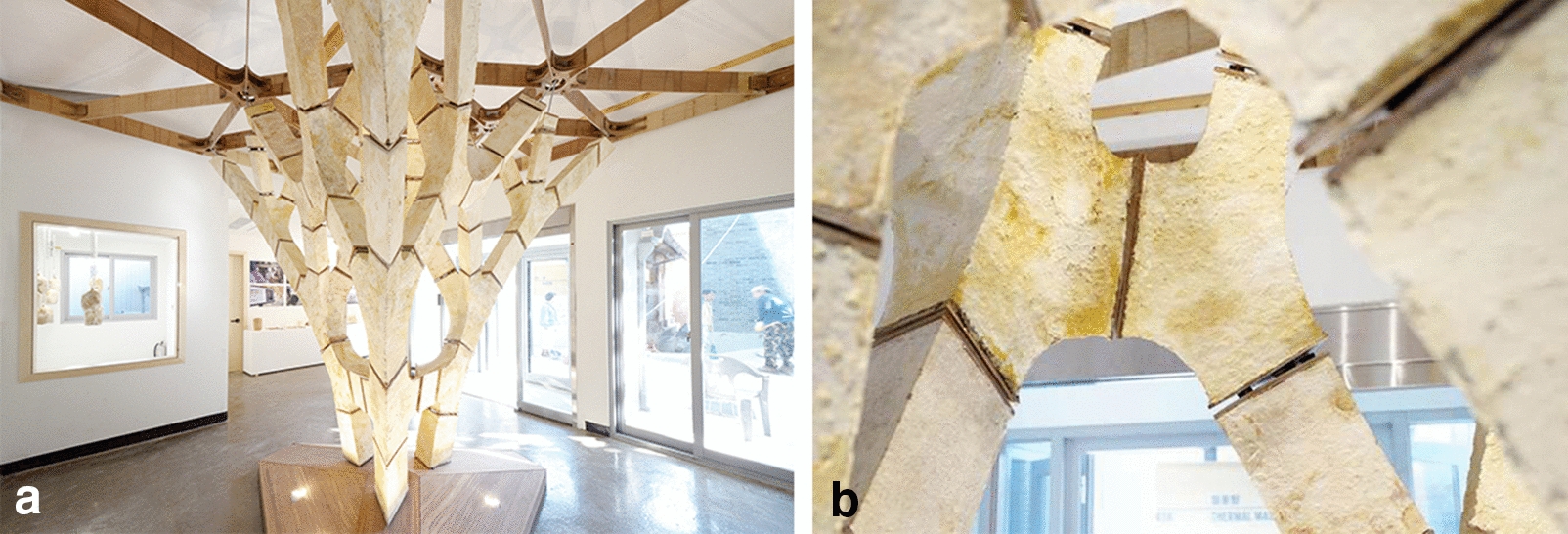
**a** Perspective of the “MycoTree” structure. **b** Close-up of “MycoTree” structure’s jointing. Credit: © Copyright ETH Zurich, Block Research Group. / Photographs by Carlina Teteris [13]

MycoTree (Fig. 4a) is a self-supporting structure that was built during the Seoul Biennale for Architecture and Urbanism as an interior installation. The project uses mycelium composites as a structural material in conjunction with digital manufacturing and parametric design. More specifically, the design of the installation used three-dimensional Graphic Statics, a structural form-finding method for generating compression-only funicular structures [12]. A joint system made of bamboo plates and steel dowels is used to compensate for the low rigidity of the material and to carry other types of forces other than mycelium-absorbed compression (Fig. 4b).

**El Monolito Micelio—Tactical Mycelium (2018)**, Georgia Institute of Technology School of Architecture/J. Dessi-Olive et al.

**Fig. 5:**
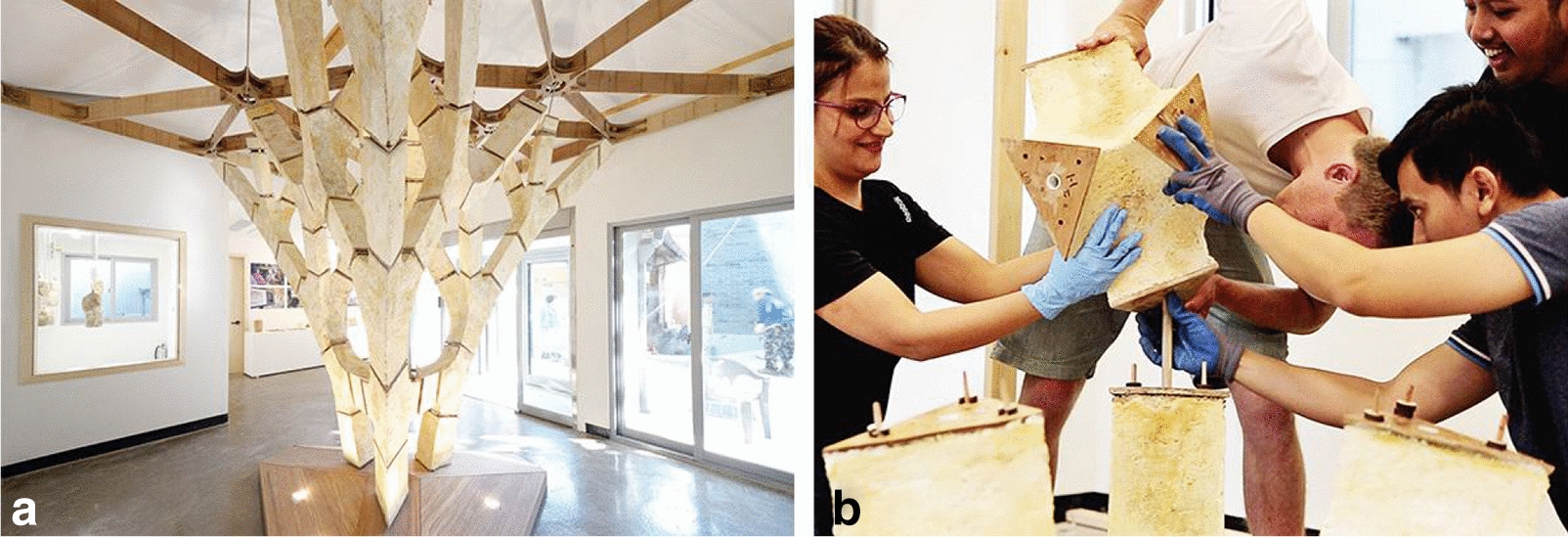
**a** Perspective of the ‘El Monolito Micelio’ structure and another mycelium composite vault from the “Tactical Mycelium” series [14]. Credit: © Jonathan Dessi-Olive. **b** The OSB supporting structural skeleton of the pavilion before the combination with the bio-composite. Credit: © Sean Miller. **c** Diagram showcasing the morphological development of the pavilion [15]. Credit: © Sean Miller

‘El Monolito Micelio’ is part of a series of mycelium construction experiments focused on vault-formed monolithic “castings” of mycelium composite materials (Fig. 5a). The Tactical Mycelium experiments borrowed construction techniques from fabric-formwork concrete casting to develop tactics for monolithic mycelium construction (Fig. 5c) and were realized with the work of students from the Georgia Tech School of Architecture under the instruction of Jonathan Dessi-Olive.

This pavilion was built for the school’s exhibition in Atlanta and used about 800 kg of mycelial composite mixture and had a size of 2.5 × 2.5 × 2.5 m. The underlying idea of the designers was to solve structural, insulation and soundproofing problems with one material to save construction time. The designers used structurally informed computational design which allowed for the mycelium composite to only absorb compressive forces [16]. A CNC-manufactured structure made of oriented strand-board (OSB) was designed for the outer shell (Fig. 5b). Holes were drilled in the outer mold to aid oxygenated mycelial growth. The mycelial mixture needed to be held in shape during the growing stage, therefore the OSB frame had to be disinfected and the mycelium composite was contained and held against the wooden frame in polypropylene geotextile. A cement mixer was used to produce a large volume of mycelial mixture on site. The most notable aspect of the project other than the monolithic casting of the mycelium composite material, was that it was left to naturally dry after the mold removal. Two weeks after the unmolding, some shrinkage and superficial cracks were noticed, due to uneven open air drying. At the end of the exhibition, the pavilion was dismantled [15].

**Growing pavilion (2019)**, Company New Heroes/P. Leboucq L. De Man et al.; Klarenbeek and Dros/E. Klarenbeek.

**Fig. 6:**
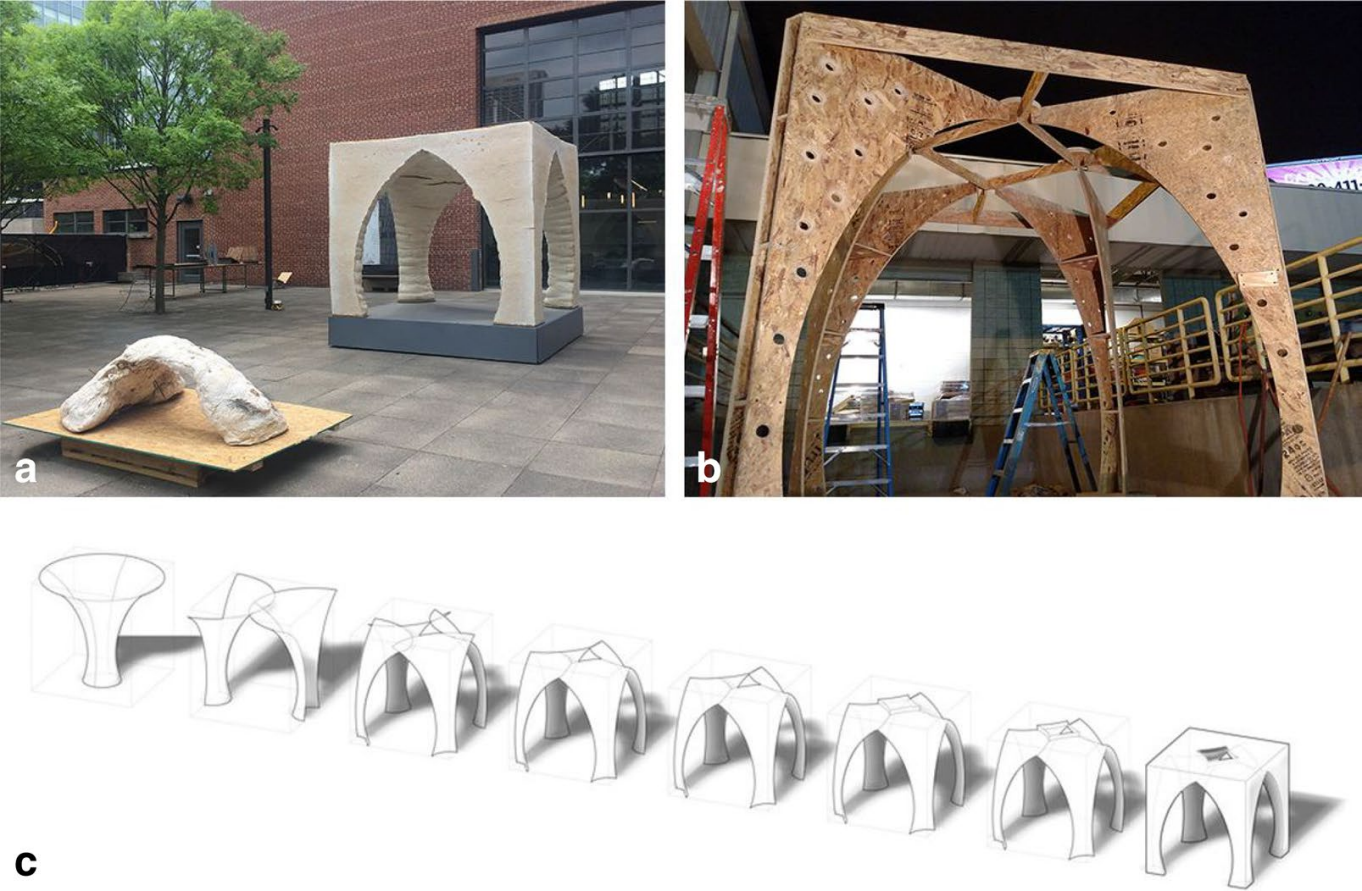
**a** Exterior view of the “Growing Pavilion” situated in Eindhoven during the 2019 Dutch Design Week. **b** Interior view and display of exhibits. Credit: © Design: Pascal Leboucq, Concept: Pascal Leboucq & Lucas De Man & Eric Klarenbeek (Klarenbeek & Dros), Initiative of: Biobased Creations and Dutch Design Foundation. / Photographs by Eric Melander [17]

The “growing” pavilion (Fig. 6a) was built as a temporary event space during the Dutch Design Week 2019. It was a collaboration of ‘Company New Heroes’, the ‘Dutch Design Foundation’ and Eric Klarenbeek that involved a plethora of companies and teams that are working on the field of circular economy. It is a study of bio-based construction for the creation of which, many different biological materials and bio-manufacturing methods were tested and used. The geometry of the pavilion is a cylindrical shape, with the main structure consisting of wooden frames and outer walls with composite mycelial panels. The panels (200 × 70 cm) manufactured by company ‘Grown’ under Ecovative’s licence (C2C Gold certification), are mounted on the wooden frame leaving exposed their sculptural surface. A wide range of biomaterials was used for the pavilion. The inner draping was made from organic cotton, Biolaminate was used as a flooring material as well as 2 types of bio-coatings (Impershield Coating and Xyhlo Coating). The coatings increased the weather resistance of the pavilion and provided a solution for a significant disadvantage of the use of mycelium composites in open-air environments. The space was used as an exhibition and performance room (Fig. 6b) and musical performances were held regularly inside the pavilion to demonstrate the sound absorption performance of mycelial materials to visitors [17]. It is worth mentioning that the creators of this pavilion have published an accompanying ‘Material Atlas’, which catalogues the life-cycle analyses of the materials used and details the fabrication, and treatment processes that were applied for the result [18].

**MY-CO SPACE (2021)**, MY-CO-X Collective/S. Pfeiffer V. Meyer et al.

**Fig. 7:**
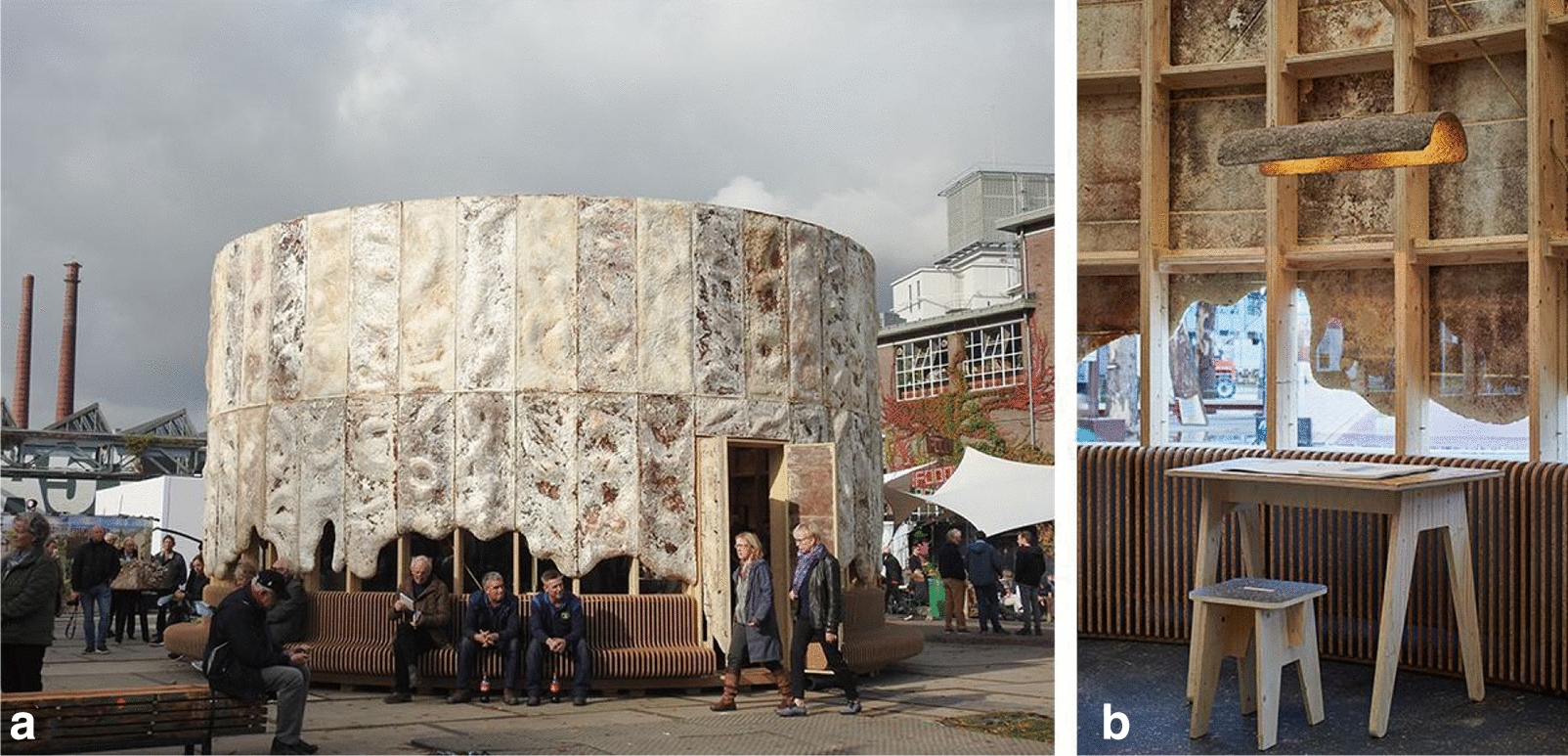
**a** Exterior view of MY-CO SPACE. Credit: © Christian Schmidts. **b** Interior view with technical equipment. Credit: © Sven Pfeiffer

MY-CO SPACE (Fig. 7a) is a collaborative project by the interdisciplinary ArtSci collective MY-CO-X founded by biotechnologist Vera Meyer and architect Sven Pfeiffer in 2020. The Berlin-based collective is comprised of artists, architects, and fungal biotechnologists. The project is a prototype for temporary dwelling for two residents and is used as a sleeping and learning station, as well as an exhibition room. Its morphology is based on the continuous functional diagram and measures 5.2 × 6.0 × 3.0 m. The structure was built as part of ‘tinyBE’, an exhibition hosting habitable sculptures in Frankfurt, Germany and references the design and functionality of manned spacecrafts. The parametrically designed prototype consists of 300 coated mycelium elements, which are attached to a substructure made of milled plywood. The substructure consists of 25 ribs which are attached to a platform and connected with horizontal wooden boards (Fig. 7b). These boards’ function both as stiffeners and as a shelving storage system. The substructure is assembled with minimal metal nuts, bolts, and angle brackets, making most of it biodegradable. The mycelium components are assembled as a lightweight protective shell enclosing the structure. The strain that was used was the basidiomycete *Fomes fomentarius* (tinder fungus), which was grown onto hemp shives and molded in pre-assembled 4.5 cm thick plywood panels. During the incubation period, the mycelium outgrew the plywood of the panel, resulting in a bound plywood-bio composite component. The panels have touching points through which the different types of forces are equally distributed into the whole surface of the shell, something that is enhanced by the different types of forces that are absorbed from the plywood and the composite.

## Conclusions

The use of fungi as a building material is an emerging field which is attracting increasing attention. In this paper we presented an array of advances in the application of fungal materials in architecture, which transform the understanding of how materials can be applied. It was our aim to present approaches that aim for a paradigm shift in the way materials are used in construction to meet the challenges in a sustainable way. However, we examine only a small part of the present activities in the field. Further relevant applications include the Urban Mining and Recycling (UMAR) unit in the research building NEST of the Swiss Federal Materials Testing and Research Institute [19], which included cultivated mycelium boards as insulation in an experimental modular building block, as well as market-ready acoustic components from the Italian company MOGU [20]. It can be observed that many architectural approaches follow a modular logic of bricks or panels of mycelial composites.

Few monolithic structures have been designed, such as the Monolito Pavilion project. Due to the plasticity of mycelium composites, monolithic approaches enable morphological free-form designs which can normally be achieved in concrete but make the material less controllable. Efforts, including bio-coating, have been made to address these problems of water resistance and durability [21]. Due to the low stiffness of mycelial composites, most projects have been reinforced with traditional building materials such as woods and metal connectors. In the MycoTree project, the material was used in a load-bearing structure, but wood and steel connectors were also used due to the limited mechanical performance of the material. A distinction can be made between production approaches for monolithic structures and those based on assemblies with discrete elements. A second distinction can be made between production approaches that kill the mycelium during the production process and those that try to preserve the mycelium as a living organism [15]. In summary, all approaches have in common that compression-based structural geometries are preferred due to the mechanical properties of the mycelial composite. Monolithic approaches to design present challenges in achieving consistent controlled production that can affect material performance, especially as the scale increases. In contrast, discrete element approaches offer greater control over production, but rely on subsequent assembly processes (Fig. 8). The heat treatment of mycelial composites to kill the mycelium provides a means of making inert material, but living material has properties that could offer new active properties such as self-healing, self-repairing, and partial self-organization. While the use of fungal-based materials can offer many advantages, the need to abolish conventional building materials completely may not be necessary. The future of construction is most likely to be an integrated process that allows architects to take advantage of both conventional and bio-based materials at the same time. Using building materials based on mycelium, new technical, aesthetic, and sustainable solutions in building are conceivable which, through biologically based functionalities such as self-regulation, adaptation, autonomous growth, and self-repair, create an alternative paradigm to the state of the art of “intelligent buildings”, which rely heavily on technical infrastructures. To introduce these materials into the construction industry, new design, planning, and construction methods are required that consider the properties of the material over the entire life cycle of a building, as well as fungal materials with precisely controllable physical properties, such as load bearing behavior, diffusion properties and fire resistances.

The developments in the field of fungal architecture are supported by expanded digital design options which have led to a far-reaching change in planning methods. Due to digital planning tools, the design language of buildings has become more diverse. Especially when working with volume materials such as mushrooms, where the components are manufactured in a “shaping” formwork process and not in a “material processing” process new, material-efficient or—for design reasons—free-form components are possible.

## Data Availability

Not applicable.
