# Correction: A review on architecture with fungal biomaterials: the desired and the feasible

**DOI:** 10.1186/s40694-022-00142-x

**Published:** 2022-07-12

**Authors:** Dimitra Almpani-Lekka, Sven Pfeiffer, Christian Schmidts, Seung-il Seo

**Affiliations:** 1 Wühlischstr. 26, 10245 Berlin, Germany; 2 Chair of Digital Design, Planning and Building, Department of Architecture, Bochum University of Applied Sciences, Am Hochschulcampus 1, 44801 Bochum, Germany; 3 Chair of Digital and Experimental Design, Department of Architecture and Urban Planing, University of the Arts, Hardenbergstr. 33, 10623 Berlin, Germany; 4 TU Berlin, Fraunhoferstraße 25, 10587 Berlin, Germany

## Correction to: Fungal Biology and Biotechnology (2021) 8:17 https://doi.org/10.1186/s40694-021-00124-5

After publication the authors became aware that they did not obtain all the necessary permission to reproduce some of the images included in the article. While the authors were able to obtain permissions for most of the figures, unfortunately this was not possible for Fig. 3 which has now been obscured. Figures 4 and 5 have been replaced with similar images of the same structures. In addition, it appears that Fig. 9 was inadvertently omitted in the main body of text. It should have been mentioned in the second paragraph of the conclusion and the article has been updated to correct this and move the Figure to a more appropriate place in the html version of the article. Furthermore, the original article stated that the ‘El Monolito Micelio’ collapsed when it was actually dismantled, and this section missed a reference to the website of Sean Miller. Finally, there were some formatting errors in the figure legends which have also now been corrected. The original article [1] has been updated.

## References

[1] Almpani-Lekka D, Pfeiffer S, Schmidts C, Seo S (2021). A review on architecture with fungal biomaterials: the desired and the feasible. Fungal Biol Biotechnol.

